# The pedagogical foundations of student voice practices: The role of relationships, differentiation, and choice in supporting student voice practices in high school classrooms^☆^

**DOI:** 10.1016/j.tate.2024.104540

**Published:** 2024-05

**Authors:** Jerusha Conner, Dana L. Mitra, Samantha E. Holquist, Enrique Rosado, Caitlin Wilson, Nikki L. Wright

**Affiliations:** a Villanova University, 800 Lancaster Ave., Villanova, PA, 19085, USA; b The Pennsylvania State University, 201 Old Main, University Park, PA, 16802, USA; c Child Trends, 7315 Wisconsin Ave., Suite 1200 W, Bethesda, MD, 20814, USA; d Conestoga High School, 200 Irish Road, Berwyn, PA, 19312, USA; e The University of Memphis, College of Education, 123 Ball Hall, Memphis, TN 38152, USA

**Keywords:** student voice, Input, Feedback, Student-teacher relationships, Interactive learning, Culturally sustaining pedagogy, Differentiated instruction, Autonomy support, Choice

## Abstract

Although teachers and administrators increasingly support the idea of student voice, questions remain about what “student voice” looks like in practice. This mixed methods study in two urban U.S. high schools explores what student voice practices in the classroom entail and how these practices relate to other pedagogical strategies. Findings reveal that student-teacher relationships, differentiated instruction, and choice serve as core building blocks for the use of student voice practices in the classroom. Findings also underscore the rarity of the student voice practices of seeking student feedback and input and engaging in collaborative decision-making with students.

## Highlights

• Classroom student voice practices (SVPs) include seeking students' feedback or input and collaborative decision-making.
• Few students report their teachers invite them to engage in collaborative decision-making.
• Effective SVPs are enabled by strong student-teacher relationships, differentiating instruction, and providing choice.
• Though qualitatively important, neither culturally-sustaining pedagogy nor interactive classrooms was associated with SVPs.

## 1 Introduction

Evidence is mounting that efforts to promote “student voice” can lead to desired academic outcomes for students. One study drawing on panel data from Chicago showed that in schools that students described as more responsive to student voice, students had better grades and attendance patterns (Kahne et al., 2022). Another study with district-wide data, found that when students felt their voice was listened to by their teachers, they reported higher affective engagement in their classes. This proxy measure of student voice was also indirectly linked to behavioral and cognitive engagement in class (Conner et al., 2022). A third study, conducted in Australia, also found significant correlations between student voice (operationalized as student participation in school decision-making) and affective engagement and cognitive engagement in school (Anderson, 2018).

While these studies build a case for the value of student voice as an intervention, the studies mentioned above also highlight ambiguity in the concept, as they each operationalize “student voice” in a different way: responsiveness to students' ideas by administrators and teachers; teachers listening to students’ ideas; and student participation in school decision-making. Reflecting this lack of conceptual precision in the research base, a recent study found that educators also understand the term “student voice” in different ways and have not coalesced around one shared meaning (Conner, 2021). Although student voice is generally understood as strategies by which students share their perspectives on their learning and education in order to inform or enact change, not all researchers or practitioners embrace this conceptualization, as evidenced by the research cited above.

The current study helps to address the conceptual murkiness in student voice by exploring three fundamental research questions: 1) What are student voice practices (SVPs), at the classroom level, that students describe experiencing and teachers report using? 2) What other instructional approaches are used by teachers who support student voice in the classroom? 3) How do these other instructional practices relate to SVPs? We pursue these questions using an exploratory sequential mixed methods approach, which combines grounded theory based on rich qualitative data with theory testing based on survey data collected in two urban high schools. Cataloging and categorizing SVPs and clarifying their relationship to other well-known pedagogical approaches can help guide practitioners — and those who prepare and educate them — to better tap into the transformative potential of student voice.

## 2 Literature review: teachers’ practice and student voice

In this section, we map the landscape of student voice in the classroom by first reviewing how scholars have conceptualized teacher practices of student voice. This section sets the stage for our first research question, which asks which SVPs students report experiencing and teachers report using. We then briefly summarize research on other teacher practices in which the term “student voice” has featured. This section helps orient our second and third research questions: What other instructional approaches are used by teachers who support student voice in the classroom, and how do these practices relate to SVPs?

### 2.1 Student voice in the classroom

Researchers distinguish SVPs at the classroom level from SVPs at the school and district levels (Mitra et al., 2021). Classroom-level SVPs, the focus of this study, can include soliciting student feedback on instructional methods, classroom policies, the curriculum, or homework and assessment practices in order to guide change; they can also entail engaging students in collaborative decision-making about these issues.

Teachers can elicit student feedback through a variety of methods, including drawing prompts, skits, and photovoice (Macbeath et al., 2003). Studying teachers across a range of grade levels and disciplines, Conner (2021) has previously found that teachers who solicit student voice report using a variety of techniques to do so, including regularly surveying or polling students and engaging students in open discussion about their teaching or “the state of the classroom.” These discussions range from formal whole class to small group discussions to informal conversations and check-ins. Students have also developed “constructive feedback forms” not only to give teachers actionable feedback “on homework, teaching styles, and engagement,” but also to improve the classroom for teachers and students alike (Gunther, 2016, p. 319).

Another feedback technique, co-generative dialogue is a method of positioning a small group of students as consultants to their teachers. It has been profitably used in the science classroom in the U.S. (Beltramo, 2017; Emdin, 2017). Co-gens, as they are colloquially called, build on a rich tradition of pupil consultation pioneered by Jean Rudduck (2007) and colleagues in the U.K. and used extensively in pedagogical partnership programs in higher education (Cook-Sather et al., 2019).

Classroom student voice can also entail collaborative decision-making, such as when students and their teacher collectively choose which topics to study (Laux, 2018) or co-design lesson plans (Basu, 2008) or classroom norms. One method of collaborative decision-making is “dot-mocracy,” in which students vote by affixing sticky dots to chart paper on matters such as curricular resources, classroom activities, or classroom rules (Mitra & Serriere, 2015). Co-created assessment rubrics, in which the teacher and students work together to determine grading criteria, offer another example of shared decision-making (Fraile et al., 2017). Youth-led participatory action research projects, action civics, problem-based learning, and service-learning approaches also frequently draw upon the language of student voice (Conner, 2015). These approaches engage students in making collective decisions about what issue or problem to study, how to study it, how to interpret and disseminate their findings, and what recommendations to make or actions to take as a result of their work together (Scorza et al., 2017). A rich repertoire of SVPs can be used by teachers in the classroom to engage students and improve their experiences as learners.

### 2.2 Student voice and other teacher practices

The language of student voice is frequently invoked in scholarship on a wide range of teacher practices, including (but not limited to) student-teacher relationships, classroom activities designed to promote active student participation, culturally sustaining pedagogy, differentiated instruction, and autonomy-support or choice. In what follows, we briefly describe each of these practices as they intersect with student voice in the literature. What becomes clear from this review is that while the language of student voice is often taken up in descriptions of certain instructional approaches, the actual SVPs and their relationship to the focal instructional framework are not always well specified (See Table 1.).

**Table 1:** Student voice in the literature on key teacher practices.

	Student-teacher relationships (STRs)	Interactive classrooms	Culturally sustaining or responsive pedagogy (CSP)	Differentiated instruction	Autonomy-Support or Choice
Student voice is	an indicator of positive STRs; it also requires and facilitates strong STRs	equated with student participation; rooted in notions of students as active, agentic learners	understood as a means to and an end goal of CSP	necessary to determine learner interests and preferences	exercised when students make choices based on their preferences and needs as learners

*Note.* STR refers to student-teacher relationships and CSP to culturally sustaining pedagogy.

#### 2.2.1 Student-teacher relationships

Student voice has been framed as an indicator of positive student-teacher relationships (STRs) as well as a practice that both requires and facilitates improved STRs. Strong STRs have been described as relationships in which students feel that their teachers value their voice (Phillippo, 2012). STRs also have been conceptualized as *precursors* to students’ willingness to engage in SVPs because strong and supportive relationships between students and teachers or school administrators help students feel comfortable voicing concerns and cultivate trust that their perspective, insights, and knowledge will be respected by adults (Biddle, 2017; Hopkins, 2022; Mitra et al., forthcoming). Additionally, enhanced STRs have been identified as *outcomes* of effective SVPs, as working collaboratively to make decisions can strengthen ties between students and adults (Benner et al., 2019; Lyons et al., 2020; Mitra, 2008).

#### 2.2.2 Interactive classroom practices

Instructional strategies that shift students from passive recipients of information to active participants in knowledge construction and learning have long been considered consonant with and conducive to student voice (Cook-Sather, 2006). Consistent with this argument, research has found that for some educators, the term “student voice” is synonymous with student participation in class (Conner et al., 2021; Holquist et al., 2023). For these educators, student voice denotes students speaking up and voicing their opinions about the material or actively engaging in groupwork and class discussions about the subject matter. Scholars, likewise, describe instructional approaches designed to promote student participation as a means of soliciting student voice. For example, Davies and Sinclair (2014) refer to Socratic questioning as a conduit for student voice, Patterson (2019) argues that student voice is a key feature of equitable groupwork, and Anthony et al. (2016) found that mixed ability groups in the math classroom “provided opportunities [for teachers] to hear the student voice” (p. 123). In her study of how teachers facilitate opportunities for student voice, Hopkins (2022) found all her case study teachers identified student-led discussions as a primary vehicle for student voice. While these conceptions of student voice as active participation may diverge from other understandings of the term (including our own understanding of classroom-level student voice as feedback or participation in collaborative decision-making), it is important to acknowledge the different ways in which the term “student voice” shows up both in the literature and among some practitioners.

#### 2.2.3 Culturally responsive and sustaining pedagogy

Both equitable, reciprocal student-teacher relationships and collaborative learning activities are defining elements of culturally relevant pedagogy, an approach to teaching minoritized students that develops their academic, cultural, and sociopolitical competence (Ladson-Billings, 1995). Over the decades since Ladson-Billings first theorized culturally relevant pedagogy, variations on the theory have been proposed, including culturally responsive pedagogy (Gay, 2000; Ware, 2006) and culturally sustaining pedagogy (Paris, 2012). Paris (2012) defines culturally sustaining pedagogy as one that “seeks to perpetuate and foster—to sustain—linguistic, literate, and cultural pluralism as part of the democratic project of schooling” (p. 95). In each of these formulations, student voice has emerged as salient (e.g., Ball, 2016; Ericson, 2021; Ferlazzo, 2016; Fink, 2019). For example, Nagle and Andrews (2019) found that project-based learning “centered on a personal learning framework and student voice” (p. 159) supported the enactment of culturally sustaining pedagogy in a middle school and a high school. Cook-Sather and Agu (2013) describe how a student voice initiative that positions students as pedagogical consultants to faculty enables both to “share authority and responsibility in developing culturally sustaining pedagogy.” In these studies, student voice is offered as a “means to” culturally responsive or sustaining pedagogy; however, Ladson-Billings (1995) originally framed student voice (or “cultural critique”) as both a means and a “goal” of CRP, as the pedagogy seeks to develop students’ capacities to critically reflect on and challenge existing inequities in education and beyond.

#### 2.2.4 Differentiated instruction

Student voice also features in some of the research on differentiated instruction, which calls for adapting instructional activities, curricular content, and forms of assessment on the basis of learners’ readiness, backgrounds, interests, and/or preferences (Tomlinson, 2017). Tomlinson (2008) identifies student voice as one of four key elements of effective differentiation, arguing, “Because differentiated instruction enables teachers to individualize so they can better respond to student needs, it provides a nurturing environment for student voice to grow” (p. 4). Although teachers may differentiate in ways that may not be visible to students (e.g., assigning students to groups based on their readiness level and giving each group a different task), the provision of choice is a hallmark of differentiation. Scarparolo and MacKinnon (2022), for example, highlight the intersections among differentiation, student voice, and choice: “The inclusion of student voice as part of differentiated instruction in this study led to the [students] reporting to be more engaged and motivated, predominantly through the amount of choice in their learning” (p. 14). Other scholars have similarly underscored the vital role student voice and choice play in differentiated instruction (Bender, 2012; Grecu, 2023).

#### 2.2.5 Autonomy-support and choice

Just as it is integral to differentiation, choice is foundational to autonomy support in the classroom. Autonomy supportive classrooms are those in which students feel able to make decisions that are aligned with their own values and aspirations (Bachmann, 2021; Reeve & Jang, 2006; Reeve et al., 2004). Autonomy supportive classrooms are often contrasted with controlling classrooms in which deadlines, external rewards, and potential punishments are used to motivate students (Stefanou et al., 2004). Instead, “autonomy support implies promoting choice, minimizing pressure to perform tasks in a certain way, and encouraging initiative” (Núñez & León, 2015, p. 278). In one study, teachers classified as autonomy supportive practiced student voice insofar as they listened to students more often and were more likely to inquire into students’ wants (Reeve et al., 1999). In her study of the instructional strategies used by teachers who embrace student voice in the classroom, Hopkins (2022) found choice to be a common practice. These educators believed that by providing choice or autonomy support, they facilitated student voice. Similarly, in a prior study, we found that when they were asked to describe their SVPs, several teachers pointed to examples of giving their students choice, including allowing students to choose which books to read and having them vote on where to go on a field trip from a set list of potential sites (Holquist et al., 2023). Choice and voice are frequently conflated by educators as well as scholars (Fitzpatrick et al., 2018; Seiler, 2011).

#### 2.2.6 Summary

Because it is seen by some as synonymous with student initiative, agency, or autonomy in the classroom, student voice aligns with a range of instructional strategies and stances that promote students’ active engagement in learning; however, as Table 1 shows, when the language of student voice is taken up in these other literatures, it is often reduced to students participating in class or availing themselves of choices that teachers have crafted for them. When understood as *student feedback on, input into, or participation in educational planning, delivery, assessment, or reform*, SVPs stand apart from these other instructional frameworks. How well such distinctions are understood by teachers or students, however, is a matter for empirical research. This study uses student and teacher perspectives on SVPs in the classroom first to theorize and then to test empirically the relationships between SVPs and other instructional approaches.

## 3 Methods

To identify the types of SVPs evident in urban high school classrooms, ascertain their prevalence, and understand their intersection with other instructional approaches, this Institutional Review Board-approved (Solutions IRB- #2021/09/20, Villanova IRB-FY2022-54), exploratory sequential mixed-methods study followed three steps (Dawadi et al., 2021). First, we collected rich qualitative data through focus groups with students, interviews with students, teachers, and administrators, and classroom observations in two large, urban, public high schools in the United States: Washington High School and Sinclair High School. Second, drawing on the themes that surfaced in the qualitative data, we developed and administered a survey to a broader sample of students at each school. Third, we used the quantitative data from the survey to test a theory of the relationships between SVPs and other instructional approaches that emerged from our analysis of qualitative data.

### 3.1 Data sources

For this study, individual interviews and focus groups followed a semi-structured protocol. They occurred in person, lasted between 30 and 60 minutes, and were transcribed and coded. They were designed to engage students in reflecting on their experiences with student voice in their classrooms and at the school. During the student interviews and focus groups, when specific teachers were mentioned who, in the students’ eyes, demonstrated support for student voice in their classroom, we followed up with those teachers, first observing and subsequently interviewing them to ascertain their views on student voice and to allow them to explain any SVPs they use that may not have been apparent in the class we observed. During classroom observations, researchers sat quietly in a corner and did not interact with participants, but took detailed notes, documenting all opportunities for student voice as well as the use of any other instructional practices. Researchers were not guided by an observation protocol; instead, they attempted to faithfully describe the classroom set up and transcribe all teacher talk, student talk, teacher instructional moves, and student responses that they could see and hear. Relevant artifacts, such as first day and end-of-course surveys, were collected from participating teachers.

Based on themes that emerged in the qualitative data, we developed and administered a survey to assess students’ experiences with and perspectives on SVPs at both the classroom and school level. At Washington High School, the survey was administered as a mandatory activity during a classroom advisory period. By contrast, at Sinclair High School, the survey was advertised on flyers around the school with a QR code.

#### 3.1.1 Measures

The survey included six classroom practice measures derived from the qualitative data and described below. The reliability and the overall measurement fit for each scale were assessed and deemed acceptable. In addition, we examined the invariance of each scale by gender, race, and family financial strain, a proxy for socioeconomic status. Information on the development and validation of the SVPs scales, including their psychometric properties and results of exploratory and confirmatory factor analyses can be found in Conner et al. (2023a). The means and standard deviations of each measure as well as the Pearson correlations among scales appear in Table 4.

For the first five classroom practice measures below, students were asked to respond to the following prompt, “How many of your teachers do the following?” Response options on a 4-point scale ranged from *None* (0) to *Most, more than half of my teachers* (3). This stem and response set invited students to reflect on practices teachers used across all their classes, rather than focusing on the practices of one particular teacher.

**Interactive Classroom.** A four-item scale was used to assess students' perceptions of their teachers’ use of strategies that promote interaction in the classroom. Using the stem, “How many of your teachers,” example items included, “encourage students to share their ideas during class” and “do activities that allow students to work together in groups.” All items were used to create a mean score. The reliability for this scale was moderate (α = 0.79).

**Differentiated Instruction.** Because teachers’ actual differentiation practices may be purposefully invisible to students, students were asked how many of their teachers ask questions of them that yield information that could serve as a basis for differentiation. The items included “ask students how they learn best,” “ask students what they want to learn about in the class,” and “ask students whether they should slow down or speed up their teaching.” These three items were used to create a mean score. The reliability for this scale was moderate (α = 0.76).

**Choice.** Students were asked about their perceptions of their teachers’ provision of choice within classrooms through a five-item scale. Items included: “allow students to choose their own topics for projects or assignments,” “let students choose the types of assignment they work on (for example, lecture, group work, games),” “give students choices for which tasks to complete for homework,” and “allow students to choose how they want to work in the classroom (for example, with a partner, with a group, alone).” All items were used to create a mean score. The reliability for this scale was good (α = 0.82).

**Input and Feedback.** Teachers' practices of seeking input/feedback were assessed with a five-item scale. Example items, following the stem, “How many of your teachers …,” included the following: “ask students what kind of assingments they want more of or less of,” “ask for students' ideas about how to make the classroom better,” and “ask for students’ suggestions about how they can get better at teaching.” All items were used to create a mean score. The reliability for this scale was strong (α = 0.90).

**Collaborative Decision-making.** A five-item scale was used to measure students' perceptions of their teachers' collaborative decision-making practices in the classroom. Example items included, “invite students to come up with classroom rules,” “work with students to plan lessons,” and “work with students to decide how students’ work should be graded.” All items were used to create a mean score. The reliability for this scale was strong (α = 0.88).

**Culturally Sustaining Pedagogy.** Students' perceptions of their teachers’ practices of culturally sustaining pedagogy were captured with a five-item scale, which included such items as “my teachers acknowledge and respect students and their backgrounds,” “my teachers encourage students to share their cultures or backgrounds,“ and “my teachers create opportunities for students to get to know others who have different cultures and backgrounds.” Items were assessed on a 4-point scale ranging from *strongly disagree* (1) to *strongly agree* (4). All items were used to create a mean score. The reliability for this scale was strong (α = 0.91).

**Student-Teacher Relationships.** A six-item measure of students’ perceptions of their relationships with teachers, adapted from a previously validated Developmental Relationships 360 measure (Pekel et al., 2018) was also included in the survey. Example items included: “my teachers show me that I matter to them,” “my teachers challenge me to be my best,” “my teachers help me accomplish tasks or goals,” “my teachers listen to my ideas and take them seriously,” “and “my teachers try to understand what my life is like outside of school.” Items were assessed on a 4-point scale ranging from *Strongly Disagree* (1) to *Strongly Agree* (4). All items were used to create a mean score (α = 0.91).

**Student demographics.** Students reported on their demographic information, including their grade level (ranging from 9th to 12th grade), their race, and their gender identity. For race, students could indicate their identity as American Indian or Native American, Asian, Black or African-American, Hispanic or Latiné, Native Hawaaian/Pacific Islander, white, biracial or multiracial, or other. Because the schools in the current sample predominantly serve students who identify as Latiné or Hispanic (62% of sample), a dummy variable for Latiné identity was created (*yes* = 1 and *no* = 0). For gender, students were asked if they identified as male, female, or non-binary. They were also asked if they identified as *cis*-gender or transgender.

**English language learner services.** The district provided administrative data on all students who participated in the survey, including whether or not students were currently receiving any English language learner (ELL) services. ELL services were measured using a dummy coded variable (*yes* = 1 and *no* = 0).

#### 3.1.2 Summary

By triangulating among the various qualitative data sources (interviews, focus groups, and observations) to build our theory, we bolstered the trustworthiness of our findings. Furthermore, the exploratory sequential mixed methods approach enabled us to pursue the mixing rationale of significance enhancement (maximizing our interpretation of the data) as well as various mixing purposes, including development, complementarity, and expansion because the survey was developed in part to explore findings that emerged in the qualitative data (Dawadi et al., 2021; Onwugebuzie & Leech, 2006).

### 3.2 School sites

The two high schools in which the data were collected are both located in a large urban district, known for its commitment to and innovations in student voice. The schools were selected by district administrators because they both had principals who strongly support student voice. During the school year that we collected data, Sinclair High School had just appointed its first Student Voice Coordinator. The Coordinator ran a weekly meeting of a Student Voice Council and worked with the Council members to conduct surveys of students regarding what could be improved at the school. An equivalent Student Advisory Board met weekly with the principal and a school counselor at Washington High School. The two schools also share similarities in the composition of their student bodies. Both schools are “newcomer schools,” welcoming recent immigrant or refugee students. Sinclair High School serves 1224 students, 87% of whom are students of color and 46% of whom qualify for free lunch. Seventy-four percent of the student body at Sinclair identifies as Latiné^1^ or Hispanic. Washington High School serves 1512 students, 81% of whom are students of color and 42% of whom qualify for free lunch. Sixty-six percent of the student body at Washington identifies at Latiné or Hispanic. Both schools report a student-to-teacher ratio of 16:1. Because these schools shared many similarities, they were well-suited for a grounded theory, comparative case study design, which aims to produce results that “might be transferred to another context and situation with similar characteristics” (Halaweh et al., 2008, p. 7).

### 3.3 Participants

In each school, we interviewed five students, conducted three focus groups with another four or five students, and interviewed one administrator and five teachers, whose classes we also observed for a period. Students were selected for participation by the Student Voice Coordinator or an administrator at each school because they represented a diverse cross-section of the student body. Certain focus groups were composed of students who represented populations of interest to the school, such as chronically absent students or English learners. In each school, approximately one-quarter of the students included in interviews and focus groups were involved in school-level student voice programs, while the others had not been directly involved. At each school, the participating teachers, who were selected because they were mentioned by one or more students as using SVPs, represented a range of disciplines and identities (See Table 2 for more information on interview participant demographics.).

**Table 2:** Interview and focus group participant demographics.

	Sinclair	Washington
Student Interviews	Black male junior Latina senior White male freshman 2 Latino seniors	2 Latina seniors White male sophomore 1 white deaf female junior 1 Latino sophomore
Student Focus Group 1	5 Latinos (1 freshmen, 2 sophomores, 2 juniors)	2 Latina (junior & senior); 1 Black female senior; 1 Latino junior
Student Focus Group 2	5 Latinas (all ELL, 1 freshman, 2 sophomores, 2 seniors)	4 Latina juniors
Student Focus Group 3	2 white male sophomores 2 Latinas (1 sophomore, 1 junior) 1 Latino sophomore	2 Latina sophomores; 2 male sophomores (1 white, 1 Latino)
Teachers Interviewed and Observed	2 Social Studies (white men) 2 Math (Latino) 1 English (white woman)	1 Social Studies (white man) 1 Math (Latino) 1 English (white woman) 2 Language (1 Latino, 1 white woman)

*Note.* ELL refers to English Language Learner.

Table 3 presents the demographic composition of the 1152 student survey respondents. Across age and gender categories, the respondents were roughly balanced. The majority of respondents identified as Hispanic or Latiné (62%), and an overwhelming share came from Washington High School. This result is a function of how the survey was administered at each school.

**Table 3:** Survey participants demographics.

	(N = 1152)
Grade	
9th	32%
10th	23%
11th	23%
12th	22%
Racial/Ethnic Identity	
American Indian	2.6%
Asian	5.9%
Black or African-American	4.3%
Hispanic or Latiné	62%
Native Hawaiian/Pacific Islander	2%
White	14%
2 or more races	7%
Gender Identity	
Female	49%
Male	47%
Non-binary	2%
*Cis*-gender	93%
Language	
English language learner	24%
Socioeconomic Proxy	
Parent attended some college	58%
School	
Sinclair	13%
Washington	87%

**Table 4:** Means, standard deviations and pearson correlations for student-teacher relationships (STRs) and classroom practices.

	Range	Mean (S.D.)	STRs	Interactive Classrooms	Culturally Sustaining Pedagogy	Differentiation	Choice	Input/Feedback	Collab. Decision-making
STRs	1–4	2.86 (0.66)	1	0.461**	0.793**	,450**	0.463**	0.467**	0.361**
Interactive Classrooms	0–3	1.98 (0.68)		1	0.453**	0.470**	0.577**	0.461**	0.303**
Culturally Sustaining Pedagogy	1–4	2.88 (0.66)			1	0.378**	0.418**	0.386((	0.282**
Differentiation	0–3	1.39 (0.87)				1	0.636**	0.747**	0.530**
Choice	0–3	1.47 (0.76)					1	0.660**	0.513**
Input/Feedback	0–3	1.22 (0.88)						1	0.622**
Collaborative Decision-making	0–3	0.91 (0.86)							1

### 3.4 Analytic approach

With the qualitative data, our analytic approach followed the principles of constructivist grounded theory (Charmaz, 1983; Strauss & Corbin, 1998). We entered each school site with a strong grounding in the literature on student voice; however, we remained open to emergent findings and surprises (Halaweh et al., 2008). Data analysis began during the collection of the qualitative data, as the four researchers met to debrief each day after conducting observations and interviews (Côté-Arsenault & Morrison-Beedy, 2005). Analysis proceeded in an iterative fashion. The team developed a rigorous coding schema, designed to capture both emergent codes and codes derived from our knowledge of the literature. This schema included 16 parent codes. Examples of parent codes were *teaching strategies; outcomes: academic; outcomes: developmental;* and *barriers or challenges to student voice.* The 12 child codes under *teaching strategies* included *checks for understanding; interactive classroom; culturally sustaining pedagogy; feedback; input;* and *collaboration,* among others. At least two members of the research team read each interview and focus group transcript, coded it according to our schema, resolved any discrepancies through conversation, and produced memos. Analyses of coded excerpts and memos, constant comparison within and across the two school cases, and attention to outliers led to preliminary findings, which were further explored in the survey data using descriptive statistics and linear regressions.

With the quantitative data, we first examined frequency scores for each of the scales to evaluate how many of their teachers, on average, students reported use each set of practices. To examine whether and how SVPs relate to the other pedagogical practices, we conducted multiple linear regressions that assess significant correlations, while controlling for other variables that may affect teachers' practices or students' perceptions of teacher practices. Each of the two sets of SVPs measures (input and feedback; collaborative decision-making) served as the dependent variables of interest. Because they had emerged as important in the qualitative data, student-teacher relationships, interactive classrooms, culturally sustaining pedagogy, differentiation, and choice served as independent variables. We controlled for school, students' grade level, their gender identity, their racial identity, and their status as English learners in the models. We included these covariates based on prior research findings of variation in students' perceptions of teacher practices by race, gender, and grade (Carvalho et al., 2014; Gentry et al., 2002; Pena-Shaff et al., 2018; Voight et al., 2015), and based on our analysis of the qualitative data, which suggested differences in students’ experiences by English Learner status.

## 4 Findings

Our findings are presented in the order of our sequential research methods and research questions. First, we discuss the SVPs that students reported experiencing and teachers reported using that emerged from the qualitative data. We then turn to the question of how these practices appeared to intersect with other instructional approaches in the classroom. We lay out the common instructional approaches that appeared to be part of the pedagogical repertoires of teachers who support SVPs, and we explain how they relate to SVPs from the vantage points of teachers and students, and from our own observations. Next, we use these qualitative findings to construct a theoretical model of the pedagogical foundation of SVPs, revealing the relationships between SVPs and other instructional approaches apparent in the qualitative data. Then, we turn to the quantitative data and share the findings from our theory testing of this model.

### 4.1 Classroom-level SVPs

#### 4.1.1 Feedback and input

Interviews with students and teachers pointed to two main types of classroom-level SVPs: *feedback* and *input*. Feedback entails sharing with a teacher what is and what is not working in a classroom. It is retrospective, based on what is currently happening or has been happening recently. It can be as quick and simple as a “check for understanding,” or as involved as an in-depth conversation or feedback form at the end of a unit or course. When asked whether and how their teachers solicited their feedback, students most commonly referred to questions their teachers asked them about the pace of class and checks for understanding. For example, one student said, “A lot of teachers do go back and they're like, ‘Hey, did you guys understand this unit? Do you need help?’” Another student referenced how his American History teacher would “ask us, ‘Do we need to slow down? Are you guys perfect with the way the pace of the classroom is moving?’” A third student described how “every time the subject changes,” one of her teacher asks, “How do you guys think I am teaching?..What could I have done better?” The student reflected that her teacher uses this feedback “to improve herself basically.”

Teachers, meanwhile, described a range of mechanisms they used to solicit feedback, including check-in's with students and written self and teacher evaluation forms or surveys. One math teacher described how he learned on a student feedback form that some students felt he had favorites. He has since tried to become intentional about treating everyone equally. Another teacher described how the feedback on an end of year survey inspired him to add more visual content to his classes. A language teacher described the analysis she does of student evaluation forms after each unit to determine whether or not to move on with the curriculum. She will reteach material if “70% of students said they still did not get the point,” even if assessments show they do. She emphatically declared, “I need the feedback.”

Input involves sharing ideas with a teacher about what could be done differently in the classroom. It is prospective, based on students' imagination or vision for the classroom. Students may give suggestions for different activities, assignments, or assessment practices. They may share what it is they would like to learn about, or they might recommend a different way of teaching them. One student recalled encountering an input type question on a history test, when the teacher asked, “How can I improve my teaching?” This question prompted the student to offer suggestions to the teacher about what he could do next year when teaching the class. Another student recalled encountering opportunities for input “at the end of the year. They’re like, ‘Hey, like, what are your suggestions that could be better for next year?’” A third student shared the powerful experience of sharing her views on an upcoming assessment with a teacher:

I've changed my government's teacher mind on many things, like presenting in front of a classroom. The first semester, I went up to him … and I told him about my experiences and why I just get so nervous and scared and jittery. And so, he ended up changing his mind for everyone, not just for me. He was like, “You know what? A student brought to my attention that it's very nerve-wracking. Students get panic attacks …. And I thought about that. So, I'm not going to make anyone present in front of the class.” So, it was really nice to have my voice heard, not just for myself, but it ended up changing his perspective … for anyone else who might deal with what I do. So that made me … I told him, “Wow, that's amazing.” And I felt like my voice was really heard.

When asked how he supports student voice in the classroom, the teacher referenced above did not mention this particular instance, but he affirmed his approach of welcoming students' ideas and being open to their suggestions. He explained that he sets his students up with some guidelines and parameters to “make choices about how you will go about doing what you're going to do. And then, if someone says, ‘I don't want to do it that way. I want to do it this way.’ And if it seems like that's a good idea, it's like, ‘Okay, let's do that!’” This teacher possessed and conveyed a high degree of receptivity to student input.

Examples of the SVPs of input and feedback were observed by the researchers or described by participants in English, Math, Social Studies, and Language classrooms, suggesting that these practices were not unique to any one discipline.

#### 4.1.2 Collaborative decision-making

In the literature on student voice, collaborative decision-making between students and teachers is often held up as the pinnacle of student voice. Across interviews and observations, we looked for such examples; however, we did not find any at either school. Although a full discussion of the perceived barriers to such joint decision-making is beyond the scope of this article, we note some of the most common reservations teachers and students shared when we pressed them on the topic of joint decision-making. Some teachers expressed reluctance to cede control in the classroom. For example, one teacher shared that she had heard of classes in which the students and teachers co-create classroom policies, but she was reluctant to use this approach. She explained, “I'm nervous each group would come up with their own set of standards, which might throw me off.” Another teacher reflected, “So I don't let them do whatever [they] want, right? But I offer: these are the two choices.” Teachers, some of whom felt constrained by the expectation to prepare students to pass state or national (Advanced Placement) and international (International Baccalaureate) exams, felt more comfortable offering students options, within parameters, than giving students free reign to decide what they learn or how they learn it.

Students also seemed perplexed by the prospect of collaborating with a teacher to make decisions about the curriculum, the classroom set up, homework, or grading and assessments, expressing the idea that it was not their place to necessarily weigh in on such matters. For some of these students, particularly the Latiné students, their deference to adult authority figures was rooted in cultural traditions. As one student commented:

Students had that sense, since [teachers are] the ones that control the room and do everything, they're just not allowed to say anything. I came from that [background]. I’m Latino, Salvadorian, and Mexican. So, my dad, he's an old type of Mexican. So, like, “This is how this is, and it's gonna stay like that.” And I never had a chance to speak out myself.

In addition to pointing out discordant cultural norms, other students acknowledged that teachers themselves sometimes had little say over the curriculum, pacing guidelines, common assessments, or classroom resources. In such cases, teachers could not very well include students in making decisions about matters over which they themselves had no authority.

### 4.2 Relationships between SVPs and other pedagogical approaches

**Fig. 1:**
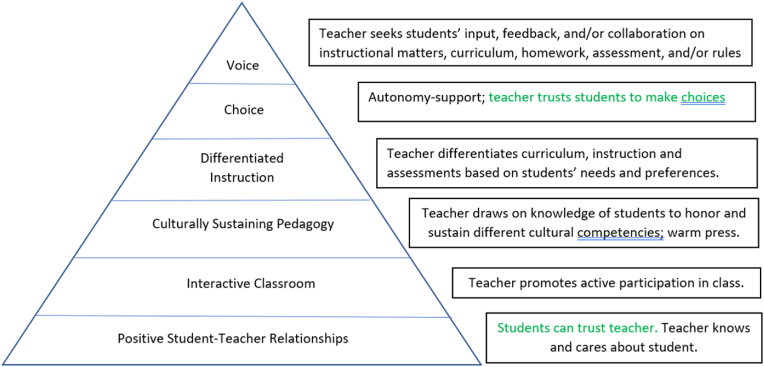
Pedagogical foundations of student voice practices.

In the classrooms of those few teachers whose students believe they support student voice (that is, the ten teachers whom we interviewed and whose classrooms we observed), five other pedagogical practices were generally apparent: intentionally fostering strong student-teacher relationships, creating interactive classrooms, practicing culturally sustaining pedagogy, differentiating instruction, and offering choice. As we discuss each of these practices below, we indicate how each undergirds the next, with the entire set serving to support SVPs at the pinnacle, as exemplified in Fig. 1. Our theorizing, therefore, posits these five practices as foundational to SVPs.

#### 4.2.1 Building student-teacher relationships

First, we noted that the teachers whom students identified as proponents of student voice were seen by their students as organized, prepared, and competent, and students relied on these qualities to determine whether or not to establish a relationship with their teacher. Students shared that they could tell which teachers spent time developing their lesson plans, and which did not, and this effort (or lack thereof) in turn affected how they related to the teacher. Students were less inclined to want to build a relationship with a teacher whom they perceived as ineffectual or indifferent, there to “just do their job and go home.” As one student shared:

You can really tell someone's vibe: if they're really trying to help you or they're just here because they're here. You know what I mean? Like [teacher name]. He's here not just because he gets paid, but because he actually wants to help us and to help better our ourselves.

Another student observed, “Some teachers, sometimes it seems like they don't care about their job. They just do [it], like, [to] try to get the money. But some teachers show enthusiasm and help us do more creative things to help us learn.” The teachers whom students singled out as proponents of student voice were all viewed as skilled, capable, and hard working.

Teachers, likewise, understood how projecting competence enabled them to gain students' trust and build strong relationships with them. One teacher explained, “To me, the real way relationships are built in the classroom is that number one, students have to feel you are competent at your job. You’re not going to build relationships with anybody if you don’t know your stuff. You can be the coolest person in the world, but if you can’t teach them, they have no use for you.” Teachers and students alike viewed professional competence as a prerequisite for relationships, which in turn proved fundamental to SVPs.

When students and teachers were asked to describe SVPs, they often emphasized the underlying relationship as a core foundation. For example, when asked how he facilitates student voice in the classroom, a math teacher reflected:

Let me see if I can think of an example … I think the surveys that I said. I think they see that I care about their opinion. And just watching their behavior. You know? Sometimes they're sad, or they look mad or something. I just pull them out and say, “Hey, what's going on?” So, I think they see that we care for them. I think that's the biggest part of it.

Teachers described how they conveyed care not just by making time for students and offering support, but also by visiting their homes, responding to emails at all hours, buying students shoes and food when necessary, and knowing their names, pronouncing them correctly, and using the students' pronouns. Some teachers used “get to know you” forms at the beginning of the year; others used icebreakers at the beginning of class, and still others expressed interest in students' interests and attended their extracurricular events. Students also highlighted these relational practices as indicative of teachers’ support for student voice. One student described how his English teacher asked the students to write a letter to her about themselves, and how he opened up to her in the letter, which enabled him to establish a stronger connection with her. Commenting on how such strong student-teacher relationships do end up impacting learning, another student offered a counter-example:

I feel like majority of teachers kind of just go off the book, and don't actually try to get a bit of an understanding of their students and their problems with learning whatever subject they are teaching, which is a bit of a problem, because it makes it harder for that teacher to teach the student if they don't understand their student.

Not only is getting to know their students and building a positive relationship with them beneficial for students' social and emotional wellbeing, but as this student indicates, it can also help the teacher to better recognize and address the students’ needs as a learner.

#### 4.2.2 Creating interactive classrooms

Strong student-teacher relationships, therefore, feed into and are reinforced by the second feature we noticed in the classrooms of teachers singled out by their students for encouraging student voice: interactive learning environments. In our observations, we saw interactive classrooms take different forms: students working in small groups on an assignment, while the teacher circulated offering support; the teacher engaging students in a lively discussion, where diverse points of view were welcomed. We even witnessed direct instruction delivered in an interactive manner, where the teacher constantly called on students, asking them to make connections between the material she was presenting and their home lives or cultures. In the most effective interactive classrooms, teachers seemed to have strong relationships with the students, using their knowledge of students to inform the interactions.

When asked about how their teachers signaled that they supported their voices, students routinely pointed to classroom activities that promoted participation. They commented on how much they valued classes that encouraged interaction not only with the teacher but also among students. One student explained that in an interactive classroom, “when I have a voice in a class, I enjoy the class a little bit more just because I feel like I'm being listened to and I'm more involved in the class. Compared to when I don't, I kind of think the class just drags along. I'm just kind of sitting there.” Teachers, too, noted how students were more engaged and less likely to “trudge through” class when collaborative groupwork and discussion replaced lecture.

#### 4.2.3 Practicing culturally responsive and sustaining pedagogy

Interactive classrooms created openings for teachers to practice culturally responsive or sustaining pedagogy. For example, a special feature of some interactive classrooms was the presence of “warm demand” or warm press. Defined as a component of culturally responsive pedagogy, warm press has been described as the teacher communicating high expectations for students, while providing clear scaffolding, support, and confidence in the students' ability to meet the expectations (Ware, 2006). In one math classroom, the teacher would ask a question and then cold call on a student, sometimes looking past the raised hands. The teacher refused to let the called upon student say, “I don't know.” Instead, he kept asking the student questions until they got to the right answer and then applauded their effort enthusiastically. In this classroom, the teacher not only knew all the students' names, but also was attuned enough to each student to know whether to keep pushing them or to let them be. To wit, this teacher checked in with one despondent student, whom he did not call on, to ask if she was okay and have a brief quiet conversation with her, and he allowed another student to sit outside in the hallway until they were ready to join the class. These examples illustrate how a strong foundation of student-teacher relationships can inform the ways in which the teacher orchestrates culturally responsive interactions in the classroom.

Beyond knowing whom to call on when, teachers can use their knowledge of students to practice other strategies consonant with culturally sustaining pedagogy (CSP). The CSP instructional moves evident in the classrooms of the teachers who were identified by students as supportive of student voice included allowing students to choose the music that played in the background, while students worked quietly together; switching seamlessly between Spanish and English in the math classroom; and referencing students' cultural backgrounds during a lesson. For example, during a lesson on clauses, a language teacher shared with students about her recent trip to Ecuador, where she ate guinea pig, and she briefly engaged the class in sharing examples of traditional cuisine from their (or their parents') home countries. Later in the lesson, when she offered an example of a sentence starting with the word “if,” she asked one student, “What is *if* in Chinese?” and another, “What is *if* in Tagalog?”, and she wrote their answers on the board. The respect and value she showed for the cultural and linguistic diversity in her classroom was clear, and she conveyed to students her desire for them to continue to honor (rather than replace or forget) their heritage and home languages. Though they only took a few seconds, these moments were indicative of culturally sustaining pedagogy. When asked about her approach to teaching, she explained the importance of getting “to know your students … I have little surveys, exercises for that. I need to know; I want to know them. It's just a sign of respect. Where are the students from? I mean, if somebody's from Honduras, then I should not say that you are from El Salvador, right?” Like many other teacher respondents, this teacher's practice of CSP was clearly rooted in her commitments to forging strong student-teacher relationships and nurturing an interactive classroom in which students felt safe contributing.

#### 4.2.4 Differentiating instruction

Some of the teachers observed also used their knowledge of their students to differentiate their instruction, again underscoring the primacy of student-teacher relationships and demonstrating how teachers can move beyond affirming students' cultural identities to incorporating curricular materials, assignments, and assessments that are responsive to cultural differences among students into their pedagogical repertoires. One student, for example, described how her English teacher allowed the students to organize themselves into different groups based on the novel they wanted to read, from a set of choices she offered them. The student, a Latina, “chose a book about a Latina who lives in America, and she gets like discriminated a lot.” The teacher's inclusion of this book as an option reflected culturally responsive differentiated instruction.

In addition to differentiating on the basis of students' identities and interests, teachers differentiated on the basis of students' needs and preferred approaches to learning. One student remarked that his teachers “all did surveys, and they were very accommodating; they would put us in little groups of who filled out what, and they would specifically help that student with what they needed.” Another student commented, “I know a lot of people are visual learners; others understand more by hearing. My math teacher was one of them that really helped me out because I'm a visual learner …. She was able to provide that for me, which I think is why I'm doing very good at math.” A third student observed: “In math, we don't all learn the same. So, he gives us different formats to do the equation, like, different functions or different ways to do it, [and you cand find what] fits you. They give us three or four, and we could use any one of them and still get the same answer.” Asked about this, the math teacher explained that he exposes students not only to different formulas but also to different note-taking methods to build students' sense of agency as learners. He shared:

Now they've been able to experience two different ways of taking notes and acquiring information. Then on that third unit, what we do is we ask them, “What do you prefer?” … Giving the kids the voice to tell us how they want to learn, I mean, it's crucial … It’s that ownership. They get to own what it is that what they're learning. They walk away saying, “We learned something today because I wanted to learn that way.”

Students and teachers alike understood how teachers' efforts to tailor their instructional approaches to meet students’ needs and preferences as learners enabled their academic success.

#### 4.2.5 Offering choice

When asked about how they promoted student voice in the classroom, many teacher respondents simply cited the provision of choice. Rather than framing choice as a tool of differentiation, they tended to discuss choice as a means of building student agency and ownership. One teacher, for example, shared her reasoning for offering students different options of learning activities in the classroom:

It's just very natural to me that I ask them if they prefer reading; if they prefer we do the activities together, or individually, or if they want to do it small groups … I respect when students say that I would rather work by myself, and I allow that. But not all the time. I still want to have that person belong to the group. But I don't feel that I need to be the boss in the classroom. I believe this is our classroom, not mine, not theirs.

Of his and his co-teacher's reasoning for offering choice, another teacher explained:

There's days, we'll approach the class, when we go, “Okay. You have two options: you can use your phones to Google the definitions, or we can spend 15 min, and we can go over the definitions. And, we leave it to a vote. And we do utilize apps, like Quizzes, and Kahoot. And we asked them, “For your review, would you rather take a practice test, or would you rather do a Quizzes or a Kahoot game?” And what we found to be successful is when we give that student choice, kids are more about it. And that's obvious, right? If I have the choice to do something, and I make that choice, now I own it.

Teachers reported that they offer students choices not only so that students become more invested and engaged in the work, but also as a means of communicating their respect for students. As one teacher explained to us, giving students some freedom to choose conveys that you trust them enough to make the right choices for themselves.

Compared to teachers, relatively few students pointed to examples of choice in the classroom, and when asked about it directly, most indicated that their teachers did not usually give them choices; however, there were a few outliers. For example, one student highlighted a recent project in his English class that allowed students different options:

For English, she assigned us a poem project where we got to go through five different websites, pick out five poems we wanted to study, and learn about them. And then we had … to create our own project ….You could paint; you could draw. It really just shows the understanding that you have of that poem and its meaning.

Other students recalled rare moments when a particular teacher gave them a choice of which unit to study, which book to read, or which classroom activities to do in a given period.

Despite students’ reticence, we did observe the provision of choice in several of the classrooms we visited. For example, in one math classroom, students had the choice to sit at low or high-top desks, and they were told they could have their work checked either by one of the two teachers or by a student who had clearly mastered the material. In a social studies classroom, the teacher welcomed students and then instructed them that they would be spending the class period investigating the gender pay gap. They had to choose two of the factors from a lengthy list to research in relation to the wage gap. They then had to represent the information they gleaned on one of three templates of their choosing. Demonstrating that they were well-acquainted with this level of self-direction and autonomy in the classroom, the students set to work right away, while the teacher circulated, providing encouragement and asking probing questions to help students deepen their analysis as needed.

#### 4.2.6 Theorizing the relationships: qualitative findings

The five sets of practices described above —building positive student-teacher relationships; creating an interactive classroom; practicing culturally sustaining pedagogy; differentiating instruction; and offering choice — appear to directly build on one another. Based on our data, we theorize that each provides a foundation for the next, with the entire set serving to undergird SVPs.

The relationships among these practices are exemplified in Fig. 1. The pyramid shape of Fig. 1 serves three functions. First, it underscores the importance of strong student-teacher relationships as the bedrock for all other practices. The first level is where students’ trust in the teacher is established. Without such trust, students are unlikely to respond to teacher overtures to have a say in the classroom. As one moves up the pyramid, with each ensuing level, the teacher demonstrates greater trust in the students, as they grant the students ever-increasing opportunities to exercise more autonomy, culminating in trusting them to use their voices constructively to inform and shape improvements in the classroom through feedback, input, or collaborative decision-making. In this way, the pyramid shows the pedagogical and relational foundations on which SVPs rest.

Second, the pyramid suggests a new pattern of practices, by indicating that each layer of the pyramid builds upon the underlying layer. Just as voice extends choice by giving students a greater say in their educational experience, choice extends differentiated instruction by going beyond the practice of grouping students on the basis of their interests, backgrounds, or readiness to empowering students to make educational choices according to what is most important to them. Similarly, differentiation extends culturally sustaining pedagogy by considering students' cultural backgrounds as one of many different factors that shape students' approaches to learning. CSP, meanwhile, extends beyond interactive classroom practices by ensuring that those interactions are rooted in respect for and understanding of students’ cultural identities. And finally, interactive classrooms assume that students will feel safe enough to participate and offer up their ideas about the material. Interactive practices, therefore, rest on the premise of strong student-teacher relationships. Without grounding in the underlying layers, any one set of practices in the pyramid would likely be less effective.

Third, the pyramid reflects the relative ubiquity of each set of practices. Based on our conversations with students, we understood interactive classrooms to be fairly common, but culturally sustaining pedagogy to be rare, differentiation and choice to be even more unusual, and SVPs of input, feedback, and collaboration to be almost unheard of. The decreasing width of each band in the pyramid signifies the declining prevalence of each set of practices.

#### 4.2.7 Testing the theory: quantitative findings

**Fig. 2:**
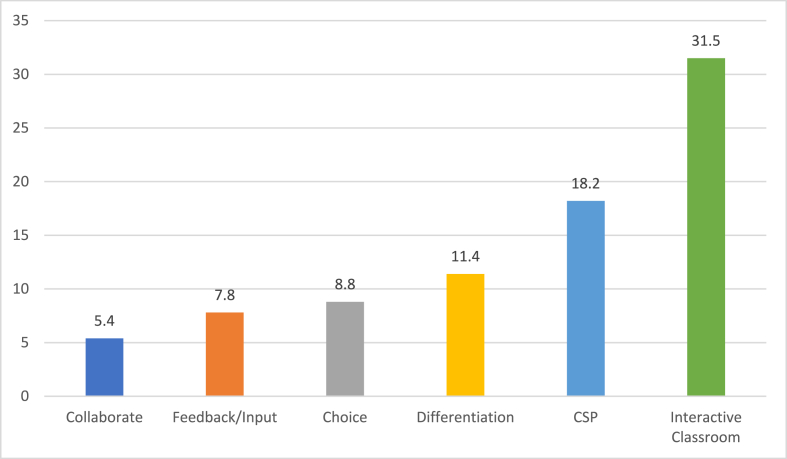
Percent of Respondents Reporting that Most/More than Half of their Teachers Perform Each Practice (*N* = 1152 students from two schools*)* *Note.* CSP refers to culturally-sustaining pedagogy.

The survey results affirmed some of the propositions we posited based on the qualitative data, while challenging others. First, consistent with our claims about the relative ubiquity or rarity of certain practices, we found from the survey data that the percentage of students who reported that their teachers used the practices associated with each level of the pyramid declined, moving upwards. Each ensuing level was less common, with SVPs the least common (See Fig. 2.). Although 81% agreed that they had strong student-teacher relationships, fewer than one-third (31.5%) believed that most of their teachers cultivated an interactive classroom, fewer than one-fifth (18.2%) agreed that most of their teachers demonstrated respect for and valuing of their cultural identities, and only 11.4% reported that most of their teachers differentiated their instruction. With only 8.8% of students reporting that most of their teachers offered them choice in the classroom, choice was almost as rare as the student voice practice of input/feedback at 7.8%. Finally, only 5% cited opportunities to engage in collaborative decision-making with most teachers. These results align with the structure and layering of the pyramid in Fig. 1.

The full regression model was a strong fit, accounting for more than 60% of the variance in the SVPs of soliciting student input and feedback. Students in higher grade levels and male-identifying students reported experiencing more teachers using these SVPs. In addition, student-teacher relationships, practices associated with differentiated instruction, and choice were positively related to teachers’ efforts to seek student input and feedback. There were no associations between these SVPs and either interactive classrooms or culturally sustaining pedagogies (See Table 5.). Although the findings about student-teacher relationships, differentiation, and choice align with our theorizing that these practices are foundational to the student voice practice of soliciting input and feedback, the lack of a significant relationship between these SVPs and either interactive classrooms or culturally sustaining pedagogy diverges from our theorized model.

**Table 5:** Regression models for student voice practices.

	Input and Feedback (Adj.R^2^ = 0.616***)			Collaborative Decision-Making (Adj.R^2^ = 0.436***)		
Predictors:	B	SE	β	B	SE	β
**School and Student Demographics**						
School	0.067	0.073	0.021	−0.098	0.086	−0.032
Grade level	0.056	0.018	0.073**	−0.012	0.021	−0.016
English Language learner	0.087	0.048	0.042	0.218	0.056	0.109***
Gender Identity						
Male identifying	0.138	0.041	0.077***	0.041	0.049	0.024
Non-binary	0.078	0.118	0.015	−0.037	0.143	−0.007
Latiné	−0.034	0.043	−0.019	0.084	0.049	0.047
**Relationships**						
Student-teacher relationships	0.142	0.055	0.102**	0.156	0.065	0.116*
**Pedagogical Practices**						
Interactive Classrooms	0.066	0.040	0.048	−0.044	0.047	−0.033
Culturally Sustaining Pedagogy	−0.062	0.051	−0.046	−0.120	0.060	−0.092*
Differentiation	0.497	0.031	0.486***	0.111	0.042	0.112**
Choice	0.343	0.038	0.293***	0.204	0.047	0.181***
Input and Feedback				0.401	0.043	0.417***

*Note.* * = *p* < 0.05, **p < 0.01, ***p < 0.001. The referent category for gender identity is female-identifying.

With regard to the student voice practice of collaborative decision-making, the fully-specified model accounted for 44% of the variance in this practice. Students identifying as English language learners were more likely to report that more of their teachers engaged them in collaborative decision-making. In addition, student-teacher relationships, practices associated with differentiated instruction, choice, and the SVPs of seeking student input and feedback were all positively related to collaborative decision-making in the classroom. Meanwhile, culturally sustaining teaching was negatively related to collaborative decision-making between teachers and students, and interactive classrooms were not associated with the practice. Again, the four positive associations converge with and support the propositions of Fig. 1 pyramid, while the negative association with culturally sustaining pedagogy and lack of association with interactive classroom diverge from our theorizing.

## 5 Discussion

This study was oriented around three research questions. First, we set out to explore the classroom-level SVPs that students reported experiencing and teachers described using at two urban high schools, well-regarded in their district for their student voice work. While the literature on student voice is replete with examples of feedback mechanisms, like questionnaires (Conner, 2021; Davis-Porada, 2023), and collaborative decision-making, like YPAR projects and co-constructed rubrics and lesson plans (Fraile et al., 2017; Scorza et al., 2017), our findings revealed that the teachers whom students named as proponents of student voice occasionally used techniques to solicit student feedback on their teaching and students’ input into educational planning, but rarely engaged students in collaborative decision-making regarding the curriculum, learning activities, homework, assessment practices, or classroom rules. These findings help clarify what SVPs look like at the classroom level, distinguishing feedback from input and both from collaborative decision-making. These distinctions add important conceptual framing and nuance to the research base.

Paired with our survey data, which tell a similar story with a wider sample of students, these findings underscore the rarity of SVPs, even in schools held up as exemplars of student voice. A great deal of prior research has observed that student voice is counter-normative and therefore, uncommon; however, the growing number of case studies of successful student voice implementation (e.g., Ball, 2016; Ericson, 2021; Hopkins, 2022; Mitra & Serriere, 2015, Nagle & Adams, 2019) may give the impression that SVPs are starting to gain traction. The school-wide survey data in this study reveals that may not be the case yet, at least at the classroom level. While there were certainly pockets of classroom-level student voice excellence in each school, from the students’ vantage point, teachers who solicited student voice in the classroom were few and far between. The qualitative and quantitative data converged on this point.

Nonetheless, teachers' and students’ accounts of the specific feedback and input techniques they have used or experienced do offer examples that educators seeking to develop SVPs might be able to adopt. Particularly because both teachers and students described these strategies as effective and impactful, they seem worthy of replication. Most of these strategies could be understood as micro-practices, such as adding a feedback question on a final exam, rather than grand interventions, like student consultant programs (e.g., Cook-Sather, 2011; Rudduck, 2007) or co-generative dialogue programs (Beltramo, 2017; Emdin, 2017), which require more sustained commitments of time and energy. The seemingly modest scope and accessibility of the SVPs found in this study may contribute to the scaling of these SVPs. However, for such micro-SVPs to be seen as effective by students, they may need to be integrated into an instructional portfolio that includes other important pedagogical commitments and practices, including differentiation and choice.

The second and third questions at the heart of this study concerned the instructional approaches that occurred in the classrooms of teachers who use SVPs and the relationships among these different pedagogical and relational strategies. In much of the literature on various instructional moves or pedagogies, such as differentiated instruction and CSP, student voice is invoked; however, the relationship between each of these approaches and the specific SVPs that complement, support, or arise from them remains unclear. Our findings help to address this ambiguity by informing the theorizing of the pedagogical foundations of student voice, represented by the pyramid in Fig. 1. This figure lays out the instructional approaches that our qualitative data suggested were necessary for effective student voice work to take hold. We found that teachers who used SVPs also showed a tendency to create interactive classrooms, offer students choice, differentiate instruction, and demonstrate culturally sustaining pedagogy (CSP), based on strong relationships with students. Other research has linked student voice to each of these practices or instructional frameworks (e.g., Hopkins, 2022; Nagle & Adams, 2019; Patterson, 2019; Scarparolo & MacKinnon, 2022; Van Aswegen, 2021), but whether student voice is a conduit to, a constituent of, or (as we argue) distinct from but complementary to these practices is not always precisely specified in the literature. This study lends greater clarity to the discussion of instructional practices by distinguishing choice, differentiation, CSP, and other strategies intended to promote student participation from the *student voice practices* of input into, feedback on, and collaborative decision-making about instruction, curriculum, assessment, and other classroom level rules.

Additionally, by drawing on both qualitative and quantitative data, we clarify the relationships among these other pedagogies and SVPs, showing which of the former serve as foundational to the latter, from the vantage point of students. Although our qualitative data suggest that all five practices undergird effective SVPs, our quantitative findings showed that student perceptions of positive student-teacher relationships, teacher practices associated with differentiation, and teachers' provision of choice were strongly related to their perception of how many of their teachers employed the SVPs of seeking student input and feedback and engaging students in collaborative decision-making. Interactive classrooms were not associated with either student voice practice, while culturally sustaining pedagogy was not linked with input and feedback and was negatively associated with collaboration. We speculate about the reasons for these findings below; however, we believe more theory testing with different student populations is needed before Fig. 1 is revised to exclude interactive classrooms and culturally sustaining pedagogy, given the strong associations between these practices and student voice, both in the literature and in our qualitative data. The lack of association between interactive classroom and SVPs in our survey results may signal students’ recognition that most of their teachers rest at this level of the pyramid. Indeed, there is a sizeable drop-off between the percent of students who report most of their teachers create interactive classrooms and the percent who report most of their teachers use SVPs. The negative association with culturally sustaining pedagogy may be the result of students not viewing collaborative decision making as culturally congruent. Because this research was conducted in schools serving predominantly Latiné student populations and because some of these students suggested to us that SVPs diverged from the cultural norms of their upbringing, it may be that students perceived teachers who were culturally affirming to be less inclined towards SVPs. Future research is needed to explore whether and how SVPs might be culturally discordant for different populations of students.

### 5.1 Limitations and future avenues for research

Other questions for future research arise from this study. For example, when SVPs are used in the absence of the theorized pedagogical foundations (such as interactive classrooms or strong student-teacher relationships), are they experienced by students as less effective? Do any of these practices moderate the relationship between student voice and student outcomes, and do such relationships vary according to students’ intersectional identities? Are particular SVPs more common in some subject areas than in others? Such questions reflect some of the limitations of the current study, including its cross-sectional survey design, its use of data from a single school district, its focus on all teachers rather than a specific classroom teacher, and its omission of variables that may lead to biased estimates, such as the number advanced or honors courses a student takes. Future research could also use more advanced statistical modeling, such as latent modeling to examine SVPs or differentiation as latent constructs, hierarchical linear regressions to account for nesting of students in classes, or structural equation modeling, to better understand the relationships among various practices.

### 5.2 Practical implications

Although it is beyond the scope of this article to explain the reasons teachers may or may not engage in SVPs, the findings of this article do raise implications for teachers and teacher educators. Specifically, teacher educators can introduce pre-service and practicing teachers to the specific instructional moves to solicit student input, feedback, and engagement in collaborative decision-making introduced in this study. These moves, which include the micro-practices of checking in with students about the pace of class, giving feedback forms at the end of a term, and asking for student input at the outset of a new unit, were cited by students and teachers alike as effective.

Additionally, this study suggests that teacher educators and administrators should continue to encourage teachers to build strong relationships with their students, differentiate instruction, and provide students with choice, as these practices provide a relational and pedagogical foundation for SVPs. A great deal of previous research has identified professional learning programs and supports that can help educators hone their relational skills (Conner et al., 2023b; Duong et al., 2019; Gehlbach et al., 2023; Kennedy & Haydon, 2021), their facility with differentiation (De Neve & Devos, 2017; Smets et al., 2020), and their approach to autonomy support in the classroom (Reeve et al., 2020). Furthermore, some research suggests that increasing teacher job satisfaction (e.g., Cheon et al., 2020), need satisfaction (e.g. Moè et al., 2022), and teacher self-efficacy (Zee & Koomen, 2020) can help teachers to become less controlling and more autonomy supportive or receptive to student voice and choice. This research raises important considerations for administrators interested in creating conditions conducive to SVPs.

## 6 Conclusion

Despite growing rhetorical support for the idea of student voice, little research has identified specific classroom-level SVPs and studied them in relation to other pedagogical strategies and frameworks. In the literature, SVPs are often conflated with participation or reduced to choice. This study advances our understanding of SVPs by providing a clear snapshot of what two specific sets of practices (input/feedback and collaborative decision-making) look like in the classroom and by revealing how they build on the relational and pedagogical practices that teacher educators have long encouraged. Our findings about the importance of strong student-teacher relationships, differentiation, and choice as core building blocks for SVPs not only provide much needed theoretical clarity for the field of student voice but also offer a roadmap for educators who wish to strengthen their instructional repertoire with SVPs and teacher educators who hope to build teachers' capacity to solicit students’ feedback, input, and collaboration in the classroom. Given mounting evidence that student voice can help generate valued academic outcomes for students (Anderson, 2018; Conner et al., 2022; Kahne et al., 2022; Mager & Nowak, 2012), and serve as a powerful driver of greater equity in schools (Lac & Mansfield, 2018), it is critical that teacher educators help teachers learn how and why to incorporate SVPs into their pedagogical portfolios. The student-centered, flexible approaches used by teachers in this study suggest the promise of a more inclusive and responsive learning environment for all learners.

## CRediT authorship contribution statement

**Jerusha Conner:** Writing – original draft, Visualization, Project administration, Methodology, Investigation, Funding acquisition, Formal analysis, Conceptualization. **Dana L. Mitra:** Writing – review & editing, Project administration, Methodology, Investigation, Funding acquisition, Conceptualization. **Samantha E. Holquist:** Writing – review & editing, Project administration, Methodology, Funding acquisition, Conceptualization. **Enrique Rosado:** Writing – review & editing, Investigation, Formal analysis. **Caitlin Wilson:** Writing – original draft, Formal analysis. **Nikki L. Wright:** Writing – review & editing, Investigation.

## Declaration of competing interest

No Generative AI or AI-Assisted Technologies were used in the writing of this manuscript. The manuscript has not been published previously, and it is not under consideration for publication elsewhere. If accepted, its publication is approved by all authors and by the responsible authorities where the work was carried out. If accepted, it will not be published elsewhere in the same form, in English or in any other language, including electronically without the written consent of the copyright-holder.

## Data Availability

The authors do not have permission to share data.

## Footnotes

1 We have chosen to use the gender-neutral term Latiné in this manuscript because Latiné is more consistent with the Spanish language than is Latinx (McGhee, 2022).

## References

[bib1] Anderson D. (2018).

[bib2] Anthony G., Hunter R., Hunter J., White B., Chinnappan M., Trenholm S. (2016). Opening up mathematics education research (proceedings of the 39th annual conference of the mathematics education research group of australasia).

[bib80] Bachmann A.M. (2021). https://hdl.handle.net/10657/8307.

[bib3] Ball C.L. (2016). Sparking passion: Engaging student voice through project-based learning in learning communities. Learning Communities Research and Practice.

[bib4] Basu S. (2008). How students design and enact physics lessons: Five immigrant Caribbean youth and the cultivation of student voice. Journal of Research in Science Teaching.

[bib5] Beltramo J. (2017). Developing adaptive teaching practice through participation in co- generative dialogue. Teaching and Teacher Education.

[bib78] Bender W.N. (2012). Project-based learning: Differentiating instruction for the 21st century.

[bib6] Benner M., Brown C., Jefferey A. (2019). https://www.americanprogress.org/article/elevating-student-voiceeducation/.

[bib7] Biddle C. (2017). Trust formation when youth and adults partner for school reform: A case study of supportive structures and challenges. Journal of Organizational and Educational Leadership.

[bib8] Carvalho C., Santos J., Conboy J., Martins D. (2014). Teachers' feedback: Exploring differences in student perceptions. Procedia: Social and Behavioral Sciences.

[bib9] Charmaz K., McCall G., Simmons J. (1983). Handbook of qualitative research.

[bib10] Cheon S.H., Reeve J., Vansteenkiste M. (2020). When teachers learn how to provide classroom structure in an autonomy-supportive way: Benefits to teachers and their students. Teaching and Teacher Education.

[bib11] Conner J. (2015). Student voice: A field coming of age. Youth Voice Journal.

[bib12] Conner J. (2021). Educators' experiences with student voice: How teachers understand, solicit, and use student voice in their classrooms. Teachers and Teaching.

[bib13] Conner J., Goldstein M., Mammen J., Hernandez J., Phillippo K., Pope D., Davidson S. (2023). What students and teachers do to build positive, reciprocal relationships: A study co-led by youth and adult researchers. American Journal of Education.

[bib14] Conner J., Mitra D., Holquist S., Wright N., Akapo P., Bonds B., Boat A. (2023).

[bib15] Conner J., Posner M., Nsowaa B. (2022). The relationship between student voice and student engagement in urban high schools. The Urban Review.

[bib16] Cook-Sather A. (2006). Sound, presence, and power: “Student voice” in educational research and reform. Curriculum Inquiry.

[bib17] Cook-Sather A. (2011). Layered learning: Student consultants deepening classroom and life lessons. Educational Action Research.

[bib18] Cook-Sather A., Agu P. (2013). Student consultants of color and faculty members working together toward culturally sustaining pedagogy. To Improve the Academy.

[bib19] Cook-Sather A., Bahti M., Ntem A. (2019). Pedagogical partnerships: A how-to guide.

[bib20] Cote-Arsenault D., Morrison-Beedy D. (2005). Maintaining your focus in focus groups: Avoiding common mistakes. Research in Nursing & Health.

[bib21] Davies M., Sinclair A. (2014). Socratic questioning in the Paideia Method to encourage dialogical discussions. Research Papers in Education.

[bib22] Davis-Porada N. (2023).

[bib23] Dawadi S., Shrestha S., Giri R.A. (2021). Mixed-methods research: A discussion on its types, challenges, and criticisms. Journal of Practical Studies in Education.

[bib24] De Neve D., Devos G. (2017). How do professional learning communities aid and hamper professional learning of beginning teachers related to differentiated instruction?. Teachers and Teaching.

[bib25] Duong M., Pullmann M., Buntain-Ricklefs J., Lee K., Benjamin K., Neuyen L., Cook C. (2019). Brief teacher training improves student behavior and student-teacher relationships in middle school. School Psychology.

[bib26] Emdin C. (2017).

[bib27] Ericson L. (2021).

[bib28] Ferlazzo L. (2016). Response: “It is long past time to meet the needs of students of color: What does culturally sustaining pedagogy look like?”. http://blogs.edweek.org/teachers/classroom_qa_with_larry_ferlazzo/2016/05/respo.

[bib29] Fink L. (2019). https://ncte.org/blog/2018/01/culturally-responsive-teaching-todays-classrooms/.

[bib30] Fitzpatrick J., O'Grady E., O'Reilly J. (2018). Promoting student agentic engagement through curriculum: Exploring the negotiated integrated curriculum initiative. Irish Educational Studies.

[bib31] Fraile J., Panadero E., Pardo R. (2017). Co-creating rubrics: The effects on self-regulated learning, self-efficacy and performance of establishing assessment criteria with students. Studies In Educational Evaluation.

[bib32] Gay G. (2000).

[bib33] Gehlbach H., Mascio B., McIntyre J. (2023). Social perspective taking: A professional development induction to improve teacher–student relationships and student learning. Journal of Educational Psychology.

[bib34] Gentry M., Gable R.K., Rizza M.G. (2002). Students' perceptions of classroom activities: Are there grade-level and gender differences. Journal of Educational Psychology.

[bib35] Grecu Y.V. (2023). Differentiated instruction: Curriculum, and resources provide a roadmap to help English teachers meet students' needs. Teaching and Teacher Education.

[bib36] Gunther R., Conner J., Rosen S. (2016). Contemporary youth activism.

[bib37] Halaweh M., Fidler C., McRobb S. (2008). Integrating the grounded theory method and case study research methodology within IS research: A possible “road map.”. ICIS 2008 Proceedings.

[bib38] Holquist S., Mitra D., Conner J., Wright N. (2023). What is student voice anyway? The intersection of student voice practices and shared leadership. Educational Administration Quarterly.

[bib39] Hopkins S.E. (2022). Passing the mic: Teachers' conceptions of student voice in urban classrooms. Impact: A Journal of Community and Cultural Inquiry in Education.

[bib40] Kahne J., Boywer B., Marshall J., Hodgin E. (2022). Is responsiveness to student voice related to academic outcomes? Strengthening the rationale for student voice in school reform. American Journal of Education.

[bib41] Kennedy A., Haydon T. (2021). Forming and sustaining high-quality student-teacher relationships to reduce minor behavioral incidents. Intervention in School and Clinic.

[bib42] Lac V.T., Mansfield K.C. (2018). What do students have to do with educational leadership? Making a case for centering student voice. Journal of Research on Leadership Education.

[bib43] Ladson-Billings G. (1995). Toward a theory of culturally relevant pedagogy. American Educational Research Journal.

[bib44] Laux K. (2018). A theoretical understanding of the literature on student voice in the science classroom. Research in Science & Technological Education.

[bib45] Lyons L., Brasof M., Baron C. (2020). Measuring mechanisms of student voice: Development and validation of student leadership capacity building scale. AERA Open.

[bib46] MacBeath J., Demetriou H., Rudduck J., Meyers K. (2003).

[bib47] Mager U., Nowak P. (2012). Effects of student participation in decision making at school: A systematic review and synthesis of empirical research. Educational Research Review.

[bib82] McGhee V. (2022). https://www.bestcolleges.com/blog/hispanic-latino-latinx-latine/.

[bib48] Mitra D.L. (2008).

[bib50] Mitra D.L., Brooks J., Heffernan A. (2021). The school leadership survival guide.

[bib51] Mitra D.L., Serriere S.C. (2015).

[bib52] Moè A., Consiglio P., Katz I. (2022). Exploring the circumplex model of motivating and demotivating teaching styles: The role of teacher need satisfaction and need frustration. Teaching and Teacher Education.

[bib53] Nagle J., Andrews W., Brinegar K., Harrison L., Hurd E. (2019). Equity and cultural responsiveness in the middle grades.

[bib55] Núñez J., León J. (2015). Autonomy support in the classroom: A review from self-determination theory. European Psychologist.

[bib56] Onwugebuzie A., Leech N. (2006). Linking research questions to mixed methods data analysis approaches. Qualitative Report.

[bib57] Paris D. (2012). Culturally sustaining pedagogy: A needed change in stance, terminology, and practice. Educational Researcher.

[bib58] Patterson A.D. (2019). Equity in groupwork: The social process of creating justice in a science classroom *cultural Studies of science education*.

[bib59] Pekel K., Roehlkepartain E.C., Syvertsen A.K., Scales P.C., Sullivan T.K., Sethi J. (2018). Finding the fluoride: Examining how and why developmental relationships are the active ingredient in interventions that work. American Journal of Orthopsychiatry.

[bib60] Pena-Shaff J.B., Bessette-Symons B., Tate M., Fingerhut J. (2018). Racial and ethnic differences in high school students' perceptions of school climate and disciplinary practices. Race, Ethnicity and Education.

[bib61] Phillippo K. (2012). You’re trying to know me: Students from non-dominant groups respond to teacher personalism. The Urban Review.

[bib62] Reeve J., Bolt E., Cai Y. (1999). Autonomy-supportive teachers: How they teach and motivate students. Journal of Educational Psychology.

[bib63] Reeve J., Cheon S.H., Yu T.H. (2020). An autonomy-supportive intervention to develop students' resilience by boosting agentic engagement. International Journal of Behavioral Development.

[bib64] Reeve J., Jang H. (2006). What teachers say and do to support students' autonomy during a learning activity. Journal of Educational Psychology.

[bib65] Reeve J., Jang H., Carrell D., Leon S., Barch J. (2004). Enhancing students' engagement by increasing teachers' autonomy support. Motivation and Emotion.

[bib66] Rudduck J., Thiessen D., Cook-Sather A. (2007). International handbook of student experience in elementary and secondary school.

[bib67] Scarparolo G., MacKinnon S. (2022). Student voice as part of differentiated instruction: Students' perspectives. Educational Review.

[bib68] Scorza D., Bertrand M., Bautista M., Morrell E., Matthews C. (2017). The dual pedagogy of YPAR: Teaching students and students as teachers. The Review of Education, Pedagogy & Cultural Studies.

[bib69] Seiler G. (2011). Reconstructing science curricula through student voice and choice. Education and Urban Society.

[bib70] Smets W., De Neve D., Struyven K. (2020). Responding to students' learning needs: How secondary education teachers learn to implement differentiated instruction. Educational Action Research.

[bib71] Stefanou C.R., Perencevich K.C., Dicintio M., Turner J.C. (2004). Supporting autonomy in the classroom: Ways teachers encourage student decision making and ownership. Educational Psychologist.

[bib72] Strauss A., Corbin J. (1998).

[bib73] Tomlinson C.A. (2008). The goals of differentiation. Educational Leadership.

[bib74] Tomlinson C.A. (2017).

[bib81] Van Aswegen J., Fovet F. (2021). Handbook of Research on Applying Universal Design for Learning Across Disciplines: Concepts, Case Studies, and Practical Implementation.

[bib75] Voight A., Hanson T., O'Malley M., Adekanye L. (2015). The racial school climate gap: Within-school disparities in students' experiences of safety, support, and connectedness. American Journal of Community Psychology.

[bib76] Ware F. (2006). Warm demander pedagogy: Culturally responsive teaching that supports a culture of achievement for African American students. Urban Education.

[bib77] Zee M., Koomen H. (2020). Engaging children in the upper elementary grades: Unique contributions of teacher self-efficacy, autonomy support, and student-teacher relationships. Journal of Research in Childhood Education.

